# Pathways to health: a framework for health-focused research and practice

**DOI:** 10.1186/1742-7622-3-18

**Published:** 2006-12-12

**Authors:** Nancy L Fleischer, Ann M Weber, Susan Gruber, Karina Z Arambula, Maya Mascarenhas, Jessica A Frasure, Constance Wang, S Leonard Syme

**Affiliations:** 1 Center for Social Epidemiology and Population Health, Department of Epidemiology, University of Michigan, 1214 South University Avenue, 2nd Floor, Ann Arbor, MI 48104-2548, USA; 2 Division of Epidemiology, School of Public Health, University of California, Berkeley, 140 Warren Hall, MC 7360, Berkeley, CA 94720-7360, USA; 3 Division of Epidemiology, Division of Community Health & Human Development, School of Public Health, University of California, Berkeley, 140 Warren Hall, MC 7360, Berkeley, CA 94720-7360 USA

## Abstract

Public health research and practice is faced with three problems: 1) a focus on disease instead of health, 2) consideration of risk factor/disease relationships one at a time, and 3) attention to individuals with limited regard for the communities in which they live. We propose a framework for health-focused research and practice. This framework encompasses individual and community pathways to health while incorporating the dynamics of context and overall population vulnerability and resilience. Individual pathways to health may differ, but commonalities will exist. By understanding these commonalities, communities can work to support health-promoting pathways in addition to removing barriers. The perspective afforded by viewing health as a dynamic process instead of as a collection of risk factors and diseases expands the number of approaches to improving health globally. Using this approach, multidisciplinary research teams working with active community participants have the potential to reshape health and intervention sciences.

## Background

The global healthcare system, which is often a "disease-care" system, is at a breaking point. Healthcare costs are escalating, and disease-prevention strategies focused on changing behaviors among individuals, one at a time, have had limited success [1,2]. Even if we were able to reduce all risk factors and cure every individual suffering from any one given disease, the occurrence of risk factors and diseases would remain nearly unchanged in the population as a whole. Disease-specific treatment strategies take focus away from the underlying causes of poor population health. The social and environmental contexts that likely produced these diseases will continue to give rise to new cases and new diseases. Our efforts to improve global health will continue to fall short of our goal [3-5].

One approach to solving this problem may be to focus on health instead of disease, and to develop comprehensive, multilevel, health-promoting policies and programs that are meaningful to people in their communities. Most public health research currently focuses on identifying risk factors for diseases one at a time at the individual level. Based on this model, we have learned a great deal about disease-specific risk factors, but little about common underlying causes of multiple diseases or overall health in populations [2]. The risk factor-based paradigm has focused most of our attention and resources on disease etiology and disease pathogenesis, and, as a result, we know little about health and the pathways to health [1,6-8].

Understanding how and why some people remain healthy is an important complementary strategy and may help advance public health research and practice. We could ask, for example, what permits 90–95 percent of people infected with tuberculosis to remain free of disease [9]? Why do most people infected with *Helicobacter pylori *never suffer any symptoms related to the infection [10]? Since smoking is the most common cause of lung cancer, how do we explain why so many smokers evade lung cancer altogether? Or finally, where should we focus our efforts with the increasing prevalence of obesity, when most obese people live fully functional lives?

In 1963 Dr. Lester Breslow observed that people are extraordinarily good predictors of their own subsequent health [11]. In the 40 years since then, his observation regarding the predictive power of self-reported health, independent of clinically-assessed objective health status, has been replicated in at least twenty-seven studies worldwide [12]. However, this line of evidence has rarely been incorporated into our disease-focused research studies. In order for people to make such accurate self-assessments, they must be taking into account a wide variety of factors in their lives and in their personal histories.

The health-focused framework we propose shifts the attention from the identification of causal factors for a single disease, one-at-a-time, to the identification of common pathways to health for people in their communities and in the full context of their lives. Raising awareness about these pathways to health has the potential to initiate useful strategies for community interventions that could contribute to sustainable improvements in health.

## Analysis

Epidemiology has its roots in infectious disease control. For decades, epidemiology has followed a model in which the goal is to find the infectious agent that is responsible for a disease and break the transmission cycle in populations. This method has brought us many advances in health, including curbing waterborne disease in post-industrialized countries, the eradication of smallpox, and the identification of HIV. However, a public health research model rooted in the pre-epidemiologic transition era is unnecessarily limited. Currently, many poor countries are facing dramatic increases in chronic diseases, sustained population growth, and an unprecedented aging boom [13,14]. While population disease patterns are transitioning from infectious diseases to chronic diseases, research models and intervention strategies have not undergone a similarly dramatic transition.

Many researchers have proposed epidemiologic theories that move beyond the risk factor paradigm. Ecologic approaches, including the ecosocial [15] and ecoepidemiology [16] frameworks, consider interactions among molecular/biological, individual, and societal factors. Others have emphasized the need for looking beyond contemporaneous, individual-level risk factors while incorporating social and environmental changes from a life-course perspective [17]. Some epidemiologists are focusing on group-level effects, including neighborhood characteristics and social capital, rather than only on individuals and their risk factors [18]. A related systems methodology suggests a "population system epidemiology," where the population is more than just a group of independent individuals [19]. Still another approach, salutogenesis, studies factors that contribute to health instead of disease [20-23]. Public health professionals are also beginning to consider strategies that shift the focus from disease to health [24-27]. Despite these advances that collectively begin to account for the complexities of health, most health researchers continue to focus on specific diseases.

As noted above, Breslow's work suggests that people understand their health in a way that cannot be explained by a purely biomedical model [11]. Individuals likely take into consideration multiple factors concurrently (including age, physical disability, current disease status, access to healthcare, social support, etc), from all levels of their life, weighing what does and does not make them healthy [12]. Based on this line of evidence, we suggest that individuals and their communities hold the key to uncovering pathways to health. Community members may offer insights on the pathways to health that surpass the collective capacity of the scientific community [28]. The integration of community-identified pathways to health in the health research agenda has the potential to produce culturally-relevant, health-focused strategies [28,29].

### A health-focused framework

We envision an organizing framework for multidisciplinary health research and practice. The framework includes external life contexts and internal multi-factorial and multi-level influences that contribute to an overall degree of health or sickness. This framework applies equally well to communities and individuals.

Drawing on multiple lines of evidence, we consider the following assumptions for this framework:

1. The human body is a dynamic complex system of interrelated physical, intellectual, and emotional components. Interpersonal relationships, communities, and the social environment are likewise dynamic complex systems.

2. The context of our lives, including our age, education, income, ethnicity, neighborhood, family, friends, job, geo-political realities, local economy, and local infrastructure can have positive or negative effects on health.

3. Stimuli enter our bodies as inputs (e.g., food, water, infectious agents, particles, smells, sounds, and events such as job loss, marriage, or acts of discrimination). On a community level, catastrophic events like floods, hurricanes, and earthquakes can also act as stimuli.

4. Filters determine how, why, when, and how much of the stimuli get into our bodies or communities. Some of these inputs enter through our conscious choice while others do not. Context actively influences the functionality of the filters, which in turn affect the impact of the inputs. For example, socioeconomic position is a common filter in determining exposure to stimuli. Where we live may influence the number of grocery stores with fresh produce that would affect our diets. Also, how much money we make may influence the type of care we can access when we are ill.

5. Inputs, which can have a positive or negative effect on health, are weighted due to external contexts and internal influences, and land on relevant balance scales. Weights are complex and dynamic and can either decay or grow and spread to other balances.

6. There are balances in our bodies and communities that determine how we cope with what enters as inputs. Each balance can be tipped towards health or towards illness. The ease with which a single balance swings between health and sickness in the face of reinforcements or assaults varies dynamically over time.

7. The position of the fulcrum on each balance in the diagram corresponds to an individual's or community's resilience or susceptibility along that continuum, and can shift over time.

8. The balances are in constant flux due to the impact of external contexts, prior inputs, or interaction with other balances. This flux makes the internal factors more likely to tip one way or another.

9. Ultimately, the dynamic accumulation of all that enters our bodies or communities over time, as well as the balances, has a net effect towards overall health or illness.

10. Every person, and every community, has a pathway to health.

**Figure 1:**
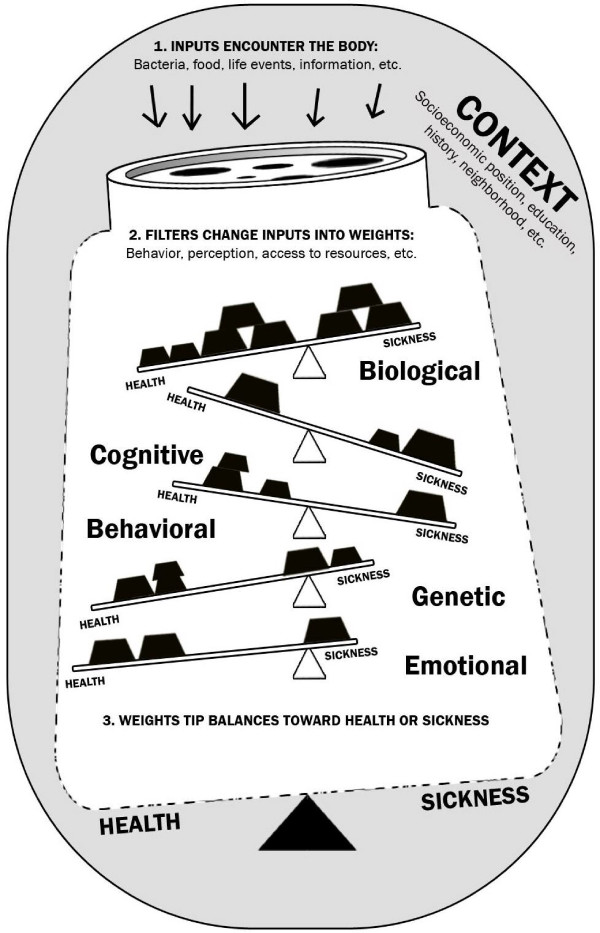
A conceptual framework for health. Context: External physical, societal environment. Inputs: External stimuli that can enter the body. Filter: Affects what enters the body. Balances: Internal facets of our lives. Affected by life contexts, inputs, history, biology. Weights: Effects of positive and negative inputs. Tip the internal balances. Dynamic: shifting, growing or lessening over time. Fulcrum: Susceptibility or resilience of a balance to weights. Dynamic: shifting over time.

The framework in the diagram below (Figure 1) represents a dynamic balance between health and illness of an individual or a community. A person or a community can be thought of as a metaphorical sum of balances, each of which can tip independently towards health or sickness. The figure explicitly demonstrates that changing contextual factors shifts the overall fulcrum and thus, the tipping point. What is not made explicit by this diagram is that there are many possible balances, that the balances interact, and that the components of the system have reciprocal influences on one another over time. This complexity is inherent in individual and community health.

A person's or a community's current state of health corresponds to the overall tilt at the base of the diagram. Long-term health, however, is a dynamic combination of: 1) the context, 2) effectiveness of the filters, 3) weights (both positive and negative) over time, 4) the rate at which new inputs enter the body, and 5) the position of each fulcrum in the system.

### Conducting health-focused research

Currently, public health research is often conducted in a community with a predetermined, disease-specific agenda. This research model, which is related to funding mechanisms and the need for clearly identified research plans, unfortunately may not match what is most important to, or has the largest impact on, the community [30]. It is unlikely that a group of public health professionals could know in advance what would make the most difference to the health of people with economic and cultural backgrounds very different from their own. However, relying on the collective community to inform the research and define its own health-related goals could instead lead to more meaningful research and more effective interventions [31-34].

We expect that employing the framework diagram and definitions presented above can be used as a tool to stimulate discussion and generate questions at the individual level. What does it mean to be healthy? What do people do to stay healthy? What do they believe will make them healthy? What prevents them from being or staying healthy? Participants can be prompted to think about diverse aspects of their lives that impact how well they feel – day to day, year to year – and how they expect to feel in the future. Compiling and analyzing information collected in this way will require combining the use of quantitative, qualitative, and innovative mixed-methods that take into account the multi-level and interactive nature of the data [7]. The analysis would ideally produce generalizable categories that capture the scope of the individual-level information on pathways to health. These individual-level results can then be used to inform group-level discussions in which the information is clarified and prioritized to adjust the individual-level pathways to reflect the community-level pathways to health. This process would integrate data from the individual level with the group level to identify representative, community-specific pathways to health. The process would ensure that the community's overall health patterns, values, and goals are reflected within the common pathways to health.

Defining a complex, health-focused framework for a specific community is a powerful first step towards improving health. Though not yet established, it is likely that when individuals and communities think about, discuss, and identify their own pathways and barriers to health, they are more likely to become advocates for pursuing these pathways and overcoming the barriers. Yet there is an essential second step: developing and implementing creative, thoughtful, and well-grounded interventions [28,35].

The framework we present is intended to guide this process by encouraging the development of locally relevant interventions designed *in concert *with the community they are intended to support [28]. Since no one person or group can address every aspect of the complex framework, the intervention design process requires collaboration among public health practitioners, policy makers, and representatives from all parts of the community in order to ensure the acceptance, feasibility, and sustainability of intervention measures [32].

The framework provides these health collaborators with four classes of complementary strategies that can motivate new directions in intervention design.

1. *Interventions can change the context by making structural, ecologic-level changes*. For example, economic incentives can encourage grocers to open stores in inner-city neighborhoods, resulting in more fruits and vegetables being available at lower cost in those areas. Likewise, building a pedestrian underpass to avoid a major roadway can minimize traffic accidents.

2. *Interventions can improve the functionality of the filters by maximizing the entry of positive inputs and minimizing the entry of the negative inputs*. For example, a free meal program in schools can provide children with nutrition while also encouraging school attendance in low-income areas. This, in turn, can lead to improved academic performance and eventually better job opportunities and healthier futures.

3. *Interventions can manipulate the impact of the weights on internal balances*. Once the inputs are in the body, we can try to minimize their negative effects or maximize their positive effects. For example, opening low-cost clinics with extended hours encourages those who normally have difficulty getting to the doctor unless it is an emergency to seek regular health-maintenance and preventive care.

4. *Interventions can adjust the position of each fulcrum to tip the balance in the direction of sustained well-being*. For example, organizing youth groups and sports leagues decreases the likelihood that youths will engage in risky behaviors, which increases resilience and fosters social capital in the communities.

Although different combinations of approaches will be suitable in different settings, being mindful that multiple options exist is invaluable in practice, especially in situations where few options seem to be available.

The framework also emphasizes and clarifies the inter-connectedness of: 1) contextual (social, political, economic, geographic) realities, 2) differences in individuals' responses, goals, and values, and 3) the overall health of the community. It is evident from the proposed framework (Figure 1) that interventions produce ripple effects throughout the entire system. Consequently, change imposed in one area may produce synergistic effects on multiple levels throughout the system causing an overall health-improving effect. Alternately, change imposed by an intervention may have a desired positive effect in one area, while negatively impacting another area. Similarly, an intervention may fail to produce a positive effect if it misses a tangential, yet crucial, aspect of health. Implementing a circumcision program to reduce HIV transmission may backfire, for example, if the extent of protection that the intervention provides is misinterpreted and circumcised men begin to engage in risky sexual behavior. Likewise, such a program may fail in traditionally non-circumcising communities if issues of cultural acceptability are not fully understood and taken into account [36,37].

The proposed framework enhances the ability of public health practitioners to anticipate indirect consequences. It also encourages careful consideration of long-term effects. For instance, since the fulcrum position represents the ease with which a community can be tipped towards or away from health, two fundamental questions emerge. First, is this intervention likely to result in a long-term effect on overall health and well-being? And second, how sure are we that the overall effect is likely to be in the intended direction, towards health?

The utility of the pathways to health framework has yet to be tested. Although testing any framework is a long-term process, we can imagine comparing the pathways to health approach to standard epidemiologic approaches through a variety of stages. Since the framework is rooted in public health practice, its success will need to be assessed in terms of its usefulness within the community.

1. The first step would be to compare the types of research questions that arise using the Pathways framework with those that arise from a disease-based approach. The framework is helpful in illustrating the types of information investigators might be interested in when considering that individuals, and communities, have latent knowledge about their health. A disease-based approach asks what risk factors individuals in the community have, while a "pathways to health" approach attempts to uncover common routes to a variety of health outcomes and obstacles along those routes.

2. If the different approaches begin with the same research question, the next step would be to see whether using the Pathways framework finds answers distinct from other methods. Under the Pathways framework, individuals, and communities, are treated as complete systems with a variety of influences and the inherent complexities. Typical epidemiologic approaches concentrate on the independent effects of various predictors. What will the difference be in the answers found from these two methods?

3. The next step would be to assess whether or not the implications for interventions vary. The proposed framework could aid researchers in communicating ideas to community members who can easily visualize weights falling on balance beams and the overall person or community teetering between ill-health and wellness. Together, communities and researchers can consider the effects that various interventions would have on different aspects of the community's health, and determine the most appropriate intervention for sustainable change. This approach can be compared to interventions that frequently target individual behavior.

4. Once research questions are asked and answered, and interventions implemented, we would need to compare whether the intervention strategies under the proposed framework were more beneficial to the community and more sustainable than those that usually occur in health research.

5. A final step for testing the new framework would be to see if, over time, health improved in the community. This could be assessed by monitoring population-based disease burden factors, including reductions in health disparities, sustainable improvements in Disability Adjusted Life Years (DALYs), and changes in the patterns of healthcare access/utilization and healthcare costs over time.

The proposed framework has a series of limitations. As a framework for health research, the Pathways framework does not lend itself to specific health outcomes or the set of predictor variables that epidemiologists have come to expect in their research. Rather, "pathways to health" are complex, multi-level systems of interconnected domains. In this context, health is a community-specific process and not an endpoint that can be defined universally for all communities. The health-focused framework highlights the urgent need for understanding health as a process. This work will require researchers to invest in defining the appropriate quantitative and qualitative techniques for investigating these pathways. It is unlikely that we will be able to use a single formula in all communities. Instead, the methods will need to be adapted to the specific context. Likewise, although we hope to be able to make connections in terms of pathways to health between communities, the process (including the appropriate questions and interventions) will be different in each. Given this, the widespread use of this framework will require investments in human capital-, research-, and community-funding.

## Conclusion

The framework provides a point of entry into a conceptual model for public health that emphasizes the identification and cultivation of pathways to health as a means to improving the human condition globally. Instead of focusing on individuals' disease/risk factor associations, we broaden our strategy for improving population health by looking at ways in which the context may be changed to shift the overall tipping point toward health. It is unlikely that the same set of pathways will work best for every person in every community. It is equally unlikely that there can be one universal definition of health. The process of determining health goals and pathways to health in populations comes from developing a shared statement of its beliefs, values, priorities, resources, and knowledge base. This approach to public health has the potential to bridge the gap between research and practice by aligning public health professionals' goals with community goals for the health of its members.

Understanding health more broadly will require a commitment to multidisciplinary collaboration. The broad impact of innovations from disciplines not traditionally associated with health is often overlooked by researchers working in disciplinary silos defined by disease category [8]. Ideally, communities would be able to identify and communicate with researchers the approaches that are aligned with their goals for population health. They could develop strategies to overcome barriers by providing structural changes to improve the community's health.

As we stumble through complexities of the tipping points between disease and health, we will confront some degree of discomfort. However, the likely insight into the health of communities has the potential to outweigh the initial discomfort and uncertainty. By working in a multidisciplinary setting with the community as an empowered partner, we stand to gain more meaningful insight into the pathways to health.

## Competing interests

The authors declare that they have no competing interests.

## Authors' contributions

The work presented in this paper was originally developed by NLF, AMW, SG, KZA, MM, and JAF as part of a Spring 2006 class at UC Berkeley School of Public Health taught by CW: Causality, Complexity and Uncertainty in Epidemiology. AMW initially conceived of the balance/fulcrum metaphor. NLF, AMW, SG, KZA, and MM participated in drafting the paper, and NLF prepared the final manuscript version. CW contributed ideas at multiple stages of the manuscript development. SLS led discussions, refined ideas, and inspired the work. All authors read and approved the final manuscript.
